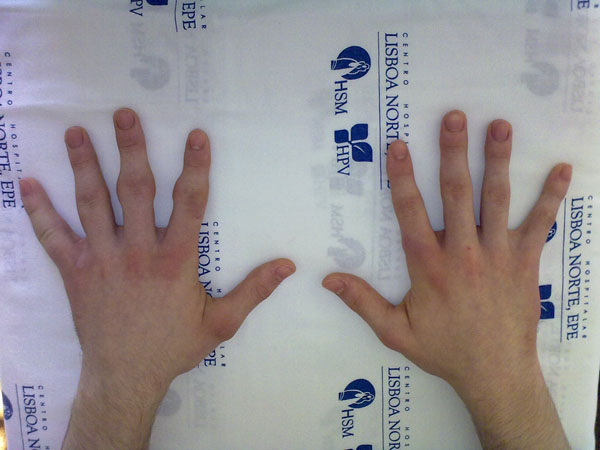# A case of pachydermodactyly in a 16 year-old male

**DOI:** 10.1186/1546-0096-9-S1-P224

**Published:** 2011-09-14

**Authors:** J Madruga Dias, M Manuela Costa, J Romeu, L Soares de Almeida, P Filipe, JA Pereira da Silva

**Affiliations:** 1 Department of Rheumatology, Hospital de Santa Maria, Lisbon, Portugal; 2 Department of Dermatology, Hospital de Santa Maria, Lisbon, Portugal

## Background

Pachydermodactyly is a rare benign superficial fibromatosis, aetiology unknown, characterized by asymmetric painless swelling of the proximal interphalangeal (PIF) joints of the hands, most frequently in adolescent males. Radiographs and MRI show only soft tissue thickening. Histological exam reveals epidermic hiperplasia, collagen fibers and fibroblast proliferation and increased mucin deposition.

## Aim

Report a case of Pachydermodactyly.

## Methods

Analysis of the patient’s clinical record.

## Results

A 16 year-old caucasian male was sent to our department due to swelling of the lateral and dorsal regions of the metacarpophalangic (MCF) and interphalagic joints of the hands, with 3 years of progression. The swelling started on the 3^rd^ PIF joint of the left hand. In 3 months all left hand PIF and MCF joints were affected. Three years later similar lesions appeared in the right hand (2^nd^ and 5^th^ PIF and MCF joints). There was no functional compromise, inflammatory signs or stiffness. The patient is otherwise healthy, without obsessive-compulsive behaviour or relevant family history. He does not practice activities requiring repetitive hand use.

On physical examination there was skin thickening in the above mentioned joints; remaining physical exam was normal.

Blood analyses (including immunological study) and hand radiographs showed no anomalies. Articular ultrasonography revealed skin thickening around previously described joints, with no sinovitis, hydrarthrosis or bone changes.

Skin biopsy showed epidermal acantosis with compact hiperkeratosis, thickening of dermis collagen fibers and increased interstitial mucin.

## Conclusion

**Figure 1:**
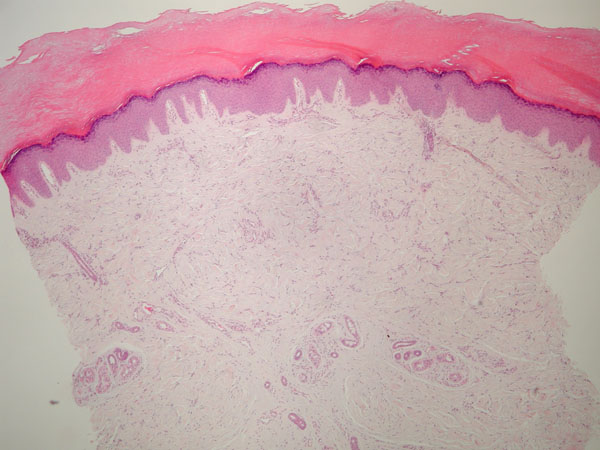


Pachydermodactyly is a rare pathology that can be mistakenly diagnosed as Juvenile Idiopathic Arthritis or Knuckle Pads syndrome. (Figure 1 and 2)

**Figure 2:**